# Uncommon Presentation of a Macrocystic Lymphatic Malformation in a Seven-Year-Old Boy: A Case Report

**DOI:** 10.7759/cureus.109788

**Published:** 2026-05-28

**Authors:** Gopika Prasad, Mc Enroe D Mordom, Sanket R Shah

**Affiliations:** 1 Pediatrics, LLH Hospital, Abu Dhabi, ARE; 2 Pediatric Surgery, Medeor Hospital, Abu Dhabi, ARE; 3 Radiology, LLH Hospital, Abu Dhabi, ARE

**Keywords:** congenital cysts of neck, cystic hygroma, infected lymphatic cyst, macrocytic lymphatic malformation, neck surgery, sclerotherapy

## Abstract

Macrocystic lymphatic malformations (old terminology: cystic hygromas) are considered a relatively rare condition; they mostly appear in the first two years of life. Cervical neck masses are a common presenting complaint in children to pediatric outpatient departments, the most common causes being infectious/inflammatory conditions of cervical lymph nodes. In this report, we aim to bring light to the fact that rarer causes like secondarily infected or hemorrhagic lymphatic malformations may also present similarly. Here, we present the case of a seven-year-old boy who had been asymptomatic before, presenting with a 10-day history of sudden-onset neck swelling associated with an upper respiratory tract infection. Treating the upper respiratory tract infection with an oral antibiotic also helped the cystic mass involute, showing that a secondary infection was the precipitating factor in the sudden increase in size of the cyst. Management options for these lymphatic malformations are between sclerotherapy and surgical excision according to indications. In this case, the size has resolved considerably with antibiotics so that the child does not require an active intervention at present and is planned to be under close radiological follow-up.

## Introduction

Lymphatic malformations are relatively rare in the pediatric population [1, 2]. Lymphangiomas are congenital lymphatic malformations that comprise about 6% of all benign lesions in infancy and early childhood [3]. Globally, the incidence of lymphatic malformations is estimated to range from one in 6,000 to one in 16,000 live births [4]. Cystic hygromas are macrocystic lymphangiomas, a type of lymphatic malformation, commonly found in the neck [1]. These macrocystic lymphatic malformations are the most frequently observed lymphangiomas in the first two years of life. Approximately 70%-80% of cystic hygromas occur in the neck, typically within the posterior cervical triangle [5]. The remaining 20%-30% may manifest in locations such as the axilla, superior mediastinum, chest wall, mesentery, retroperitoneal region, pelvis, and lower limbs [6]. Lymphatic malformations are present at birth in about 50% of affected newborns, and 90% of cases appear by the age of two [7]. Histologically, there are three types of lymphangiomas: capillary, cavernous, and cystic. Cystic hygromas involving the neck are thought to arise secondary to failure of the jugular lymph sacs to join the lymphatic system, resulting in tiny sac-like structures sprouting from the existing cystic space. Lymph-like fluid is secreted into these endothelial-lined cystic spaces. Thus, local dilation and enlargement of the cystic spaces will be the result. Lymphatic malformations often become apparent following a sudden increase in size due to infection or intralesional bleeding, which can lead to an initial appearance in the neck [8]. Spontaneous decompression or reduction in size is rare.

Macrocystic lymphatic malformations can masquerade as cervical lymphadenopathy because both may present as soft, painless, and occasionally fluctuant swellings in the neck. Accurate differentiation is critical for proper management, as misdiagnosing them as enlarged lymph nodes can delay appropriate interventions. In this context, we describe an unusual case of a seven-year-old boy presenting with left lateral neck swelling initially resembling lymphadenitis, which turned out to be a cystic hygroma with secondary infection and bleeding. Although there are reports of lymphatic malformations presenting later in life, such occurrences are quite rare. Despite their rarity, lymphatic malformations should be considered a significant differential diagnosis for lateral neck swellings at any age.

## Case presentation

**Figure 1:**
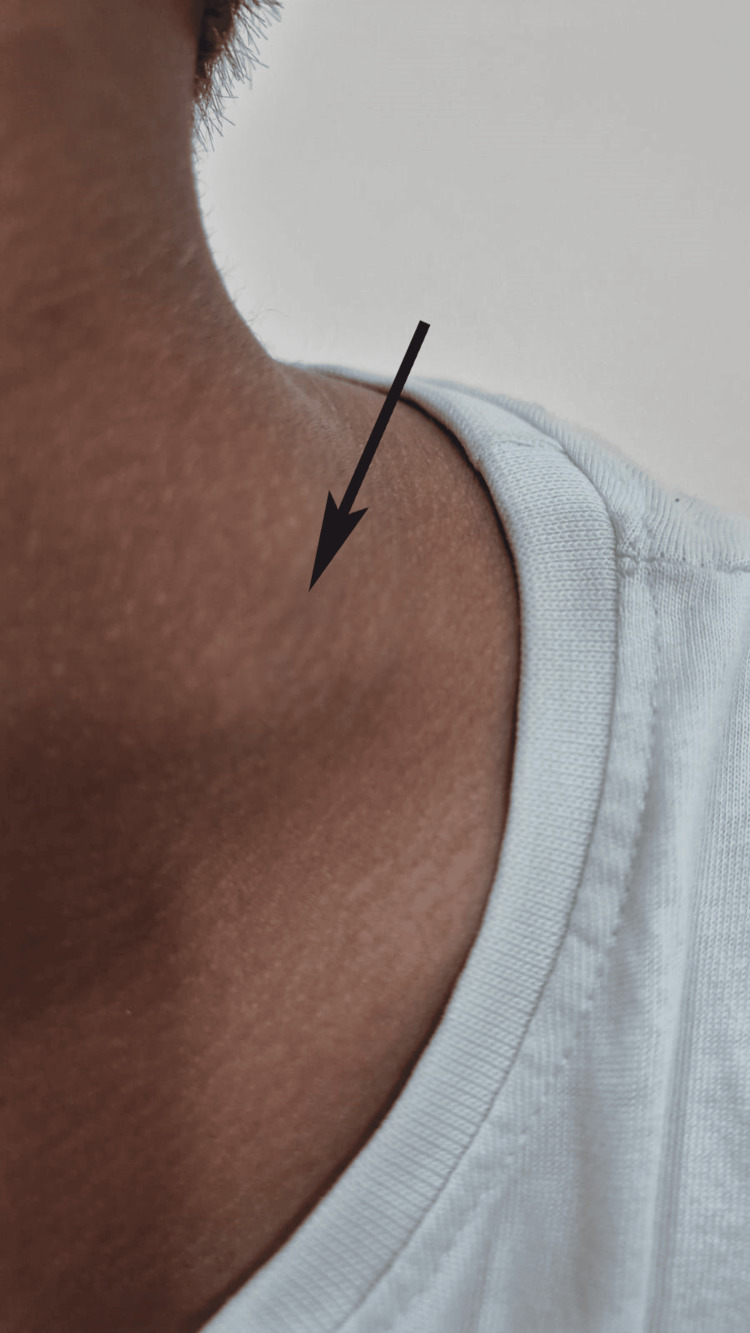
Swellings seen on the side of the neck The swellings were firm, fluctuant, and tender. One swelling measured 4.5 × 2 cm above, and another measured 2 × 2 cm below the first one.

A seven-year-old boy with a sudden onset of neck swelling noted on the lower part of the left side of the neck for the past 10 days, along with a cough and low-grade fever. The child had completed a course of oral azithromycin from another hospital with a diagnosis of cervical lymphadenitis. There was no reduction in the swelling even after the course of azithromycin. On examination, the swelling was firm and fluctuant, of the size of 4.5 x 2 cm, with another swelling of 2 x 2 cm below the first one. The swellings were tense, elastic, and tender; however, there was no erythema or warmth to account for cervical lymphadenitis or abscess formation (Figure 1). Transillumination was doubtful, as there was a thin rim of a halo visible. Throat examination showed signs of pharyngitis.

Investigations

**Figure 2:**
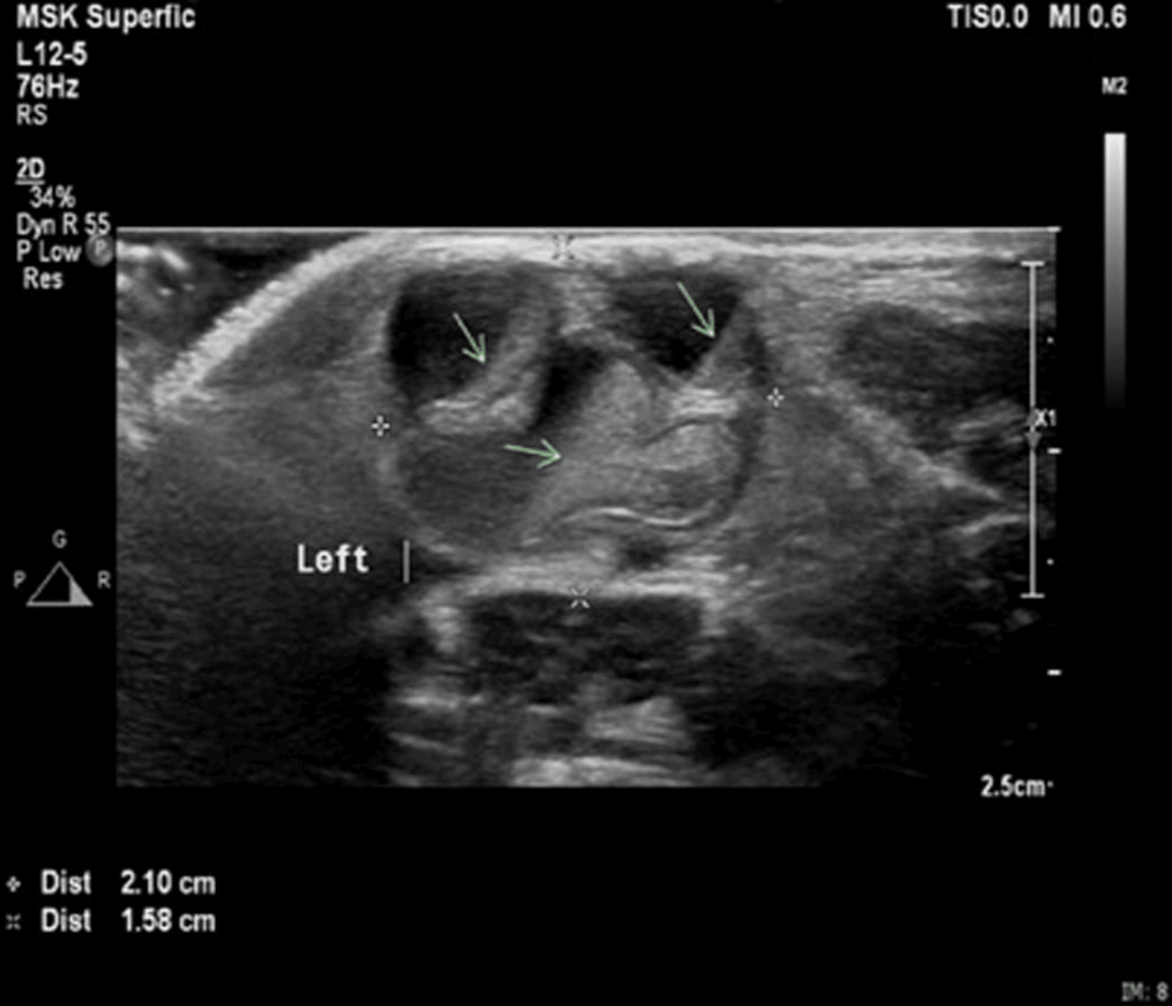
Ultrasound image of the swelling on the left side of the neck A grayscale ultrasound image taken at the level of the left posterior triangle of the neck showing a well-defined oval-shaped multiloculated cystic lesion (marked by calipers), revealing multiple fluid-debris levels (marked by arrows). Debris seen in the dependent part of each locule represents blood products/hemorrhagic contents. Note the lack of surrounding inflammatory changes.

**Figure 3:**
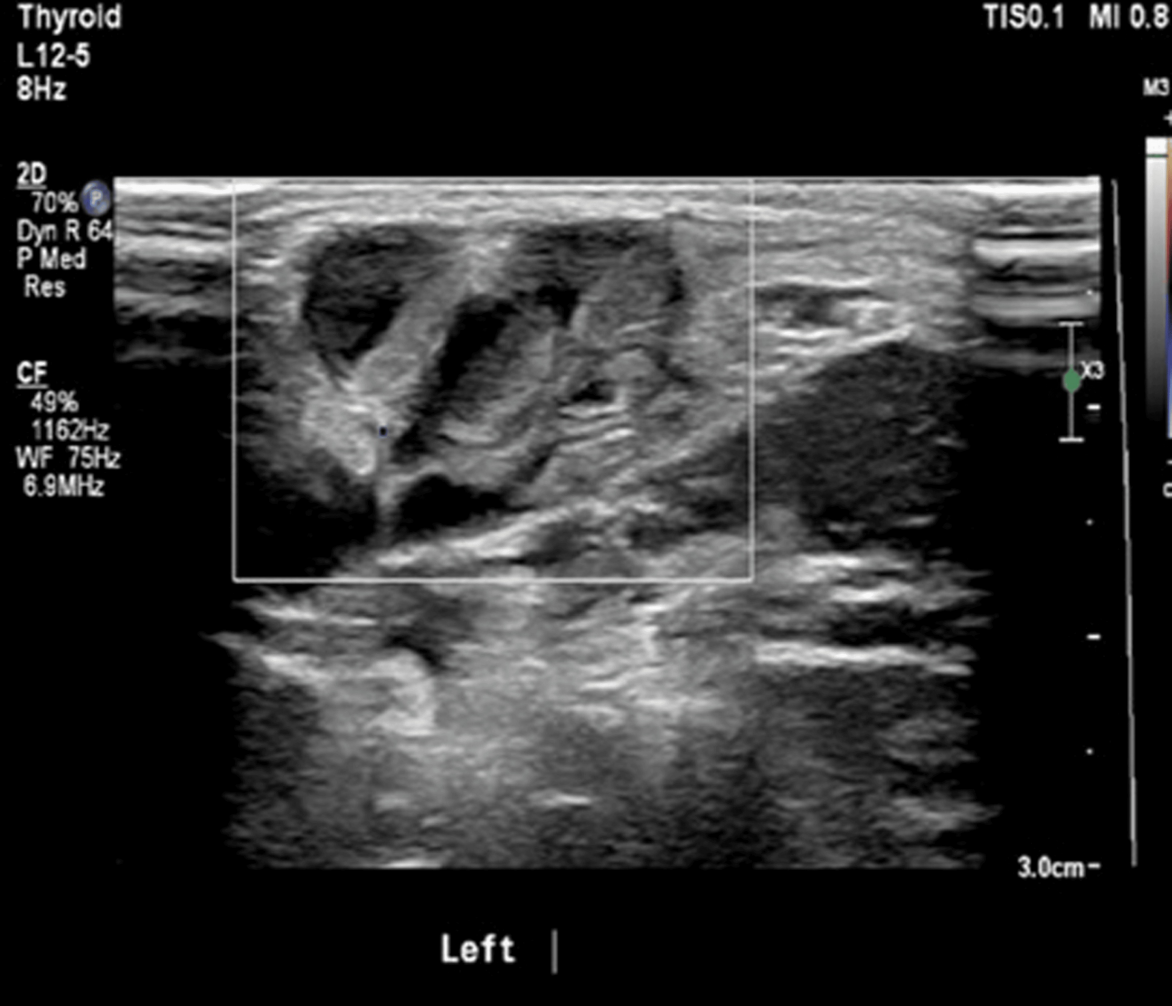
Color Doppler image of the swelling on the left side of the neck A color Doppler image obtained at the same level shows no obvious intralesional blood flow, suggesting a nonvascular origin of this lesion. Also note the lack of peripheral vascularity, which excludes the diagnosis of necrotic cervical lymph node/abscess.

An ultrasound scan of the neck was done, which showed the diagnosis of lymphatic malformation with hemorrhage within. The ultrasound of the neck showed a well-defined, oval-shaped, multiloculated but interconnected cystic lesion of size 2.1 x 1.6 x 2.6 cm seen in the soft tissues of the posterior triangle of the neck on the left side, showing multiple fluid-debris levels, with indentation over the left external jugular vein suggestive of lymphatic malformation with hemorrhage within. No obvious intralesional vascularity was noted on color Doppler. No obvious perilesional inflammatory changes were noted (Figures 2-3).

The blood counts and inflammatory markers, including C-reactive protein, were normal (white blood cell count: 6900 cells/µL, neutrophils: 41%, lymphocytes: 48%, and C-reactive protein: 0.6 mg/dL), probably because the child was already on azithromycin. With the highest possibility of a recent increase in swelling of the cystic hygroma induced by an upper respiratory tract infection, it was decided that the child receive a course of a broad-spectrum antibiotic like co-amoxiclav, which would clear the lymphangioma of any seedings from the infection site.

**Figure 4:**
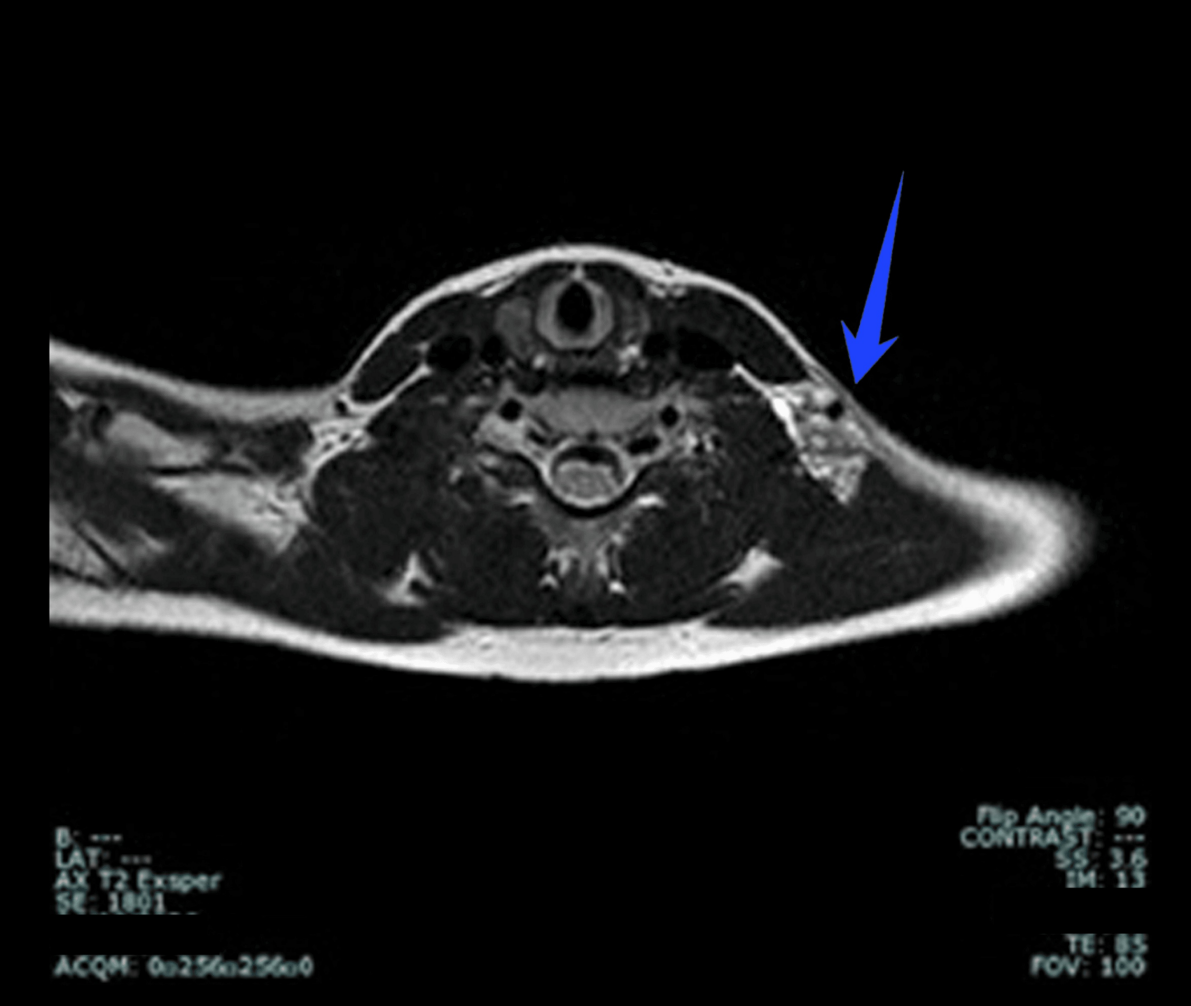
Axial T2-weighted MRI image of the neck Axial T2-weighted image of the neck showing an oval-shaped multiloculated cystic lesion (marked by arrow) in the left posterior triangle of the neck showing multiple fluid-debris levels without surrounding inflammatory changes.

**Figure 5:**
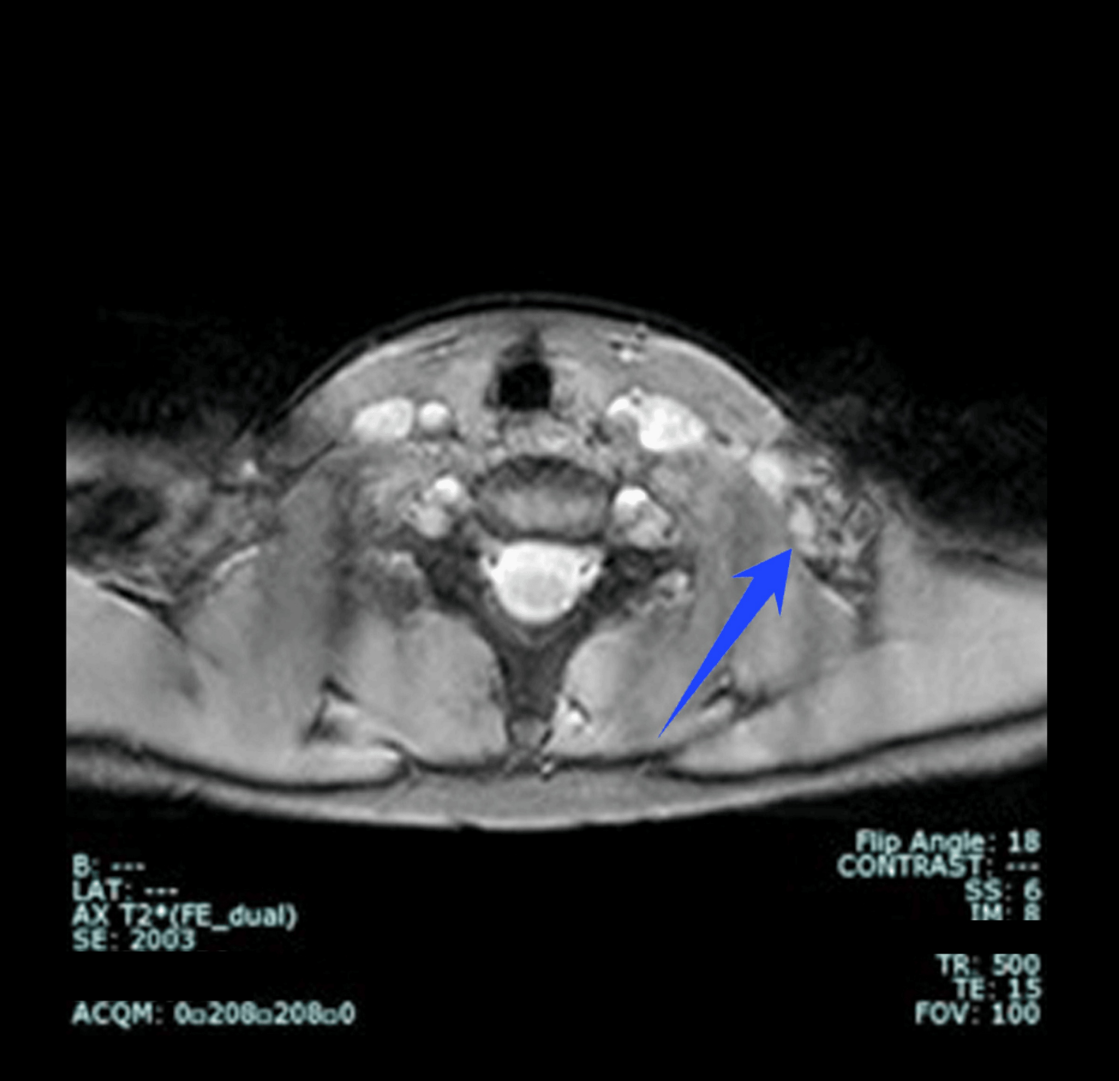
Axial T2 gradient echo image of the neck An axial T2 gradient echo image of the neck showing blooming and a hyperintense signal within the dependent debris in multiple loculi of the cystic lesion in the left posterior triangle of the neck, confirming the presence of blood products/hemorrhage within the lesion.

**Figure 6:**
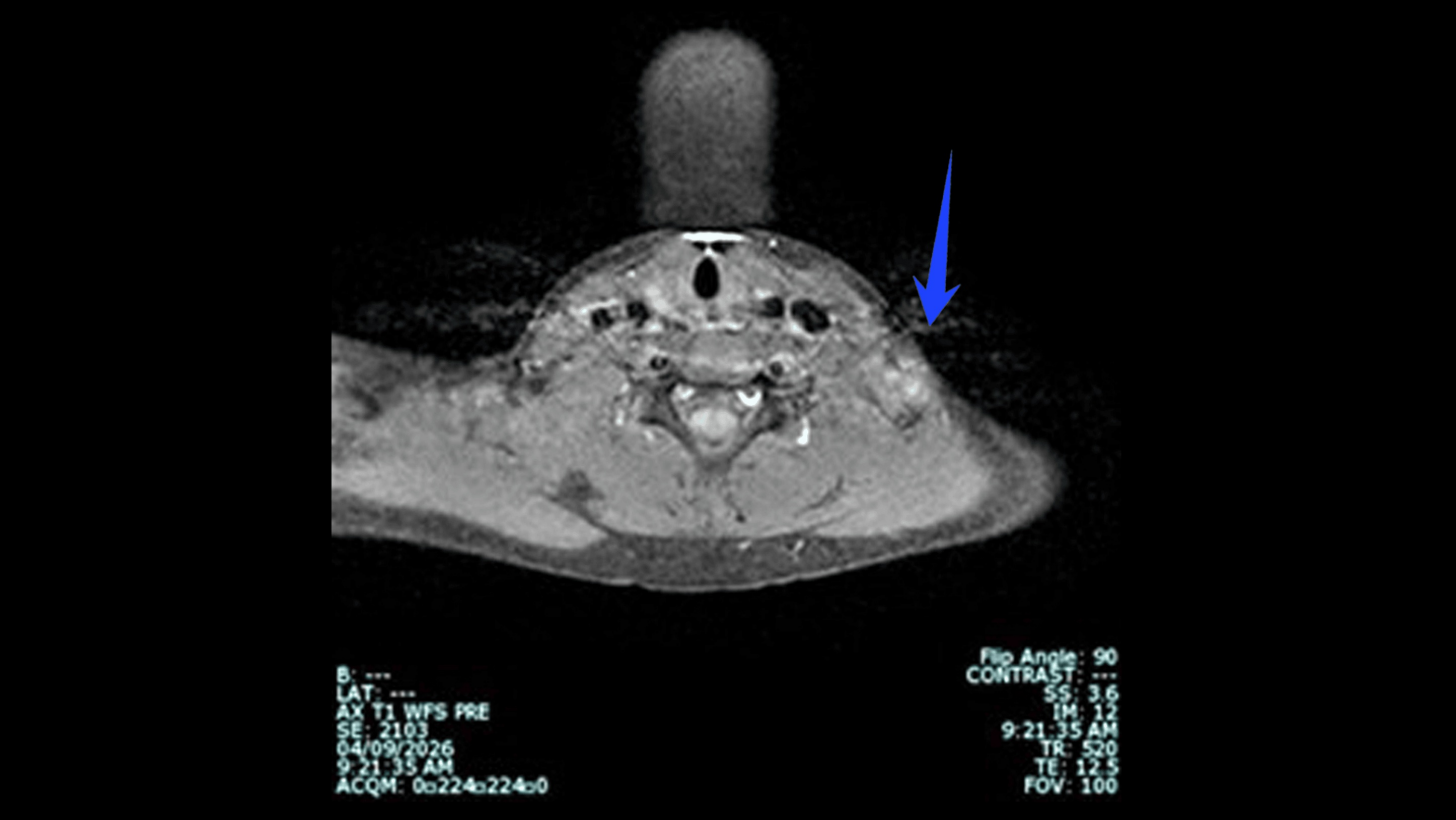
Axial T1-fat saturated image of the neck Axial T1 fat-saturated image of the neck showing blooming and hyperintense signal within the dependent debris in multiple loculi of the cystic lesion in the left posterior triangle of the neck, confirming the presence of blood products/hemorrhage within the lesion.

**Figure 7:**
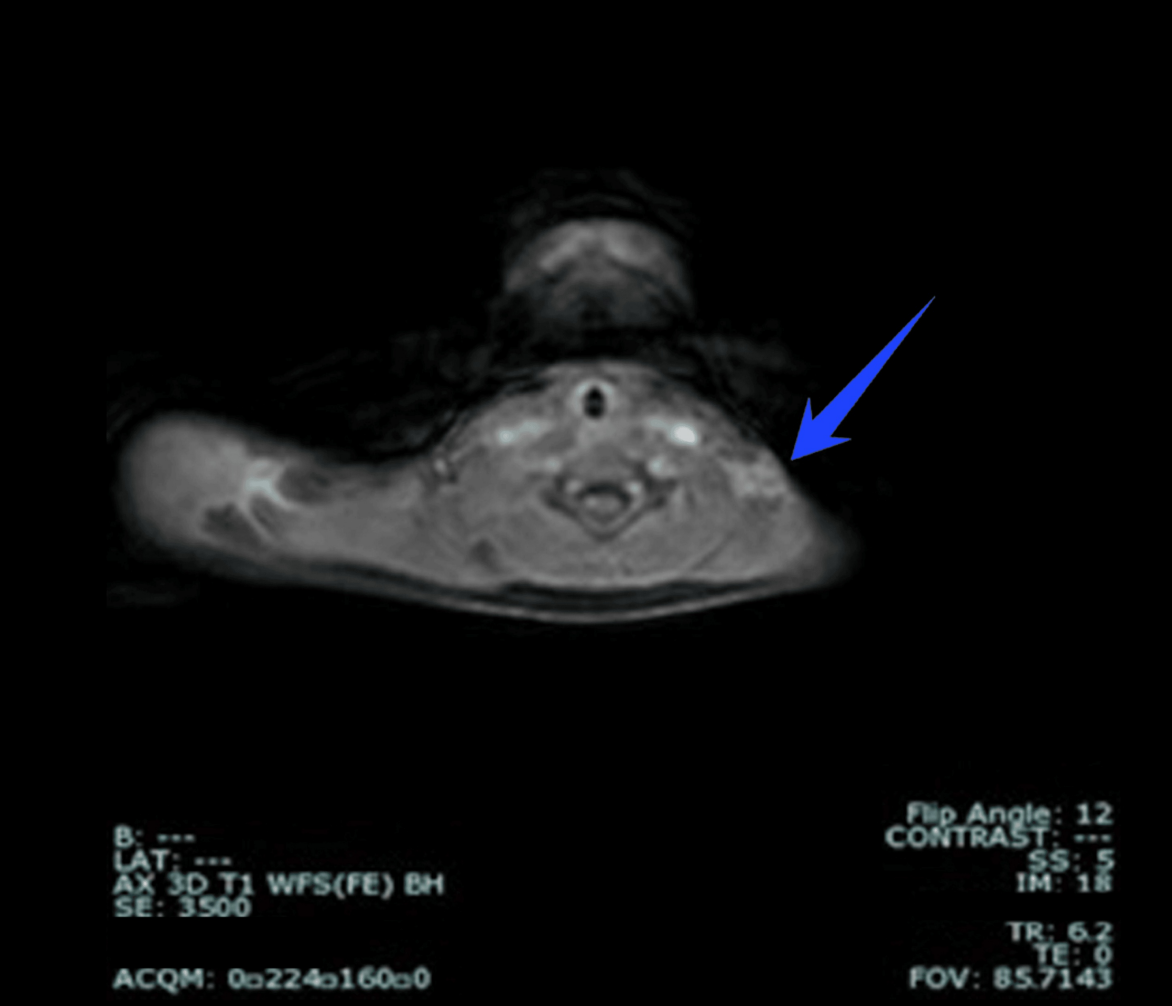
Axial post-contrast T1-weighted fat-saturated image of the neck Axial post-contrast T1-weighted fat-saturated image of the neck showing mild subtle peripheral rim enhancement to the oval-shaped multiloculated cystic lesion in the left posterior triangle of the neck.

Meanwhile, an MRI of the neck with contrast was arranged, which confirmed the diagnosis. The findings were suggestive of a well-defined, benign-appearing cystic lesion of approximate size 3.3 cm x 1.7 cm x 3 cm in the posterior triangle of the neck on the left side, showing multiple loculations and fluid debris levels containing hemorrhagic contents in these multiple cystic loculi (Figures 4-7).

After starting co-amoxiclav, the size of the swelling reduced significantly. By day 3, the swelling had become non-tender and had reduced in size. By day 6, the swelling was only 1.5 x 1.5 cm.

The swelling has considerably reduced in size with oral antibiotics, suggesting that the recent increase in size, firmness, and tenderness of the cystic hygroma was due to infection. The small size of the swelling at present does not warrant a surgical exploration at present. Considering the small size of the swelling, the age of the child, the close proximity to major neck vessels and vital structures, and the possible chances of future recurrence of the lymphangioma, it is decided in the best interest of the child that, at present, he does not need a surgical resection or a sclerosing therapy. The child is planned for follow-up every three months with ultrasonography. In the future, if there is a rapid increase in size not attributable to any recent infection, the child will be considered for sclerotherapy or surgical resection by the pediatric surgeon after discussion with the parents.

## Discussion

Lymphatic malformations are clusters of endothelial-lined spaces that contain lymphatic fluid and can vary widely in size, from tiny capillary-like channels to large cysts several centimeters across [9]. These cysts develop when the lymphatic vessels become partially or completely blocked during early fetal development, specifically around the sixth week of gestation. This blockage disrupts the normal connection between the lymphatic and venous systems, resulting in the formation of cystic spaces. While some cysts are single-chambered (unilocular), most are divided into multiple chambers (multilocular), all containing lymphatic fluid [10]. Experts believe these malformations arise from leftover embryonic lymphatic tissue capable of ongoing growth. As a result, they can enlarge by sprouting new vessels and may extend across anatomical regions [11]. Lymphangiomas, a type of lymphatic malformation, are benign and classified by imaging into three categories: macrocystic (over 2 cm in diameter), microcystic (under 2 cm), and mixed forms [12, 13].

Lymphatic malformations are benign and typically present at birth or, most often, within the first two years of life. They can be detected prenatally. Sixty-one percent of lymphatic malformations are linked to aneuploidy, with trisomy 21 being the most common if a cystic hygroma is identified in the first trimester and Turner syndrome if detected in the second trimester [14]. In the absence of aneuploidy, there is a significant risk of association with cardiac abnormalities, such as coarctation of the aorta and hypoplastic left heart syndrome. Lymphatic malformations may also be part of genetic syndromes such as Noonan syndrome [15].

A distinctive characteristic of lymphatic malformations is their susceptibility to infections, which may lead to cellulitis or more widespread illness. Infections elsewhere in the body or viral illnesses can also cause lymphatic malformations to increase in size and tension. The primary source of infection is often the respiratory tract, although primary infection of the lymphatic malformations can occur as well. Cystic components of lymphatic malformations are prone to bleeding within the lesion, especially following trauma or due to abnormal venous connections. Vesicles involving the skin may leak thin, blood-tinged fluid or present as red, purple, or black nodules [16].

These tumors typically present as soft, painless masses that are usually translucent. If infection occurs, the cystic hygroma may enlarge, become warm, red, and tender, and the patient may develop a fever. The infection can affect either the entire lymphatic malformation or just a few cysts. During active infection, the lesion may lose its transluminescent quality. Occasionally, a lymphatic malformation can develop into an abscess that requires drainage to relieve symptoms [17].

Cysts appearing later in life may be attributed to delayed proliferation of residual lymphatic tissue or secondary factors such as infections, trauma, or iatrogenic causes [17].

The posterior triangle of the neck is the most frequent location for macrocystic lymphatic malformations. These lesions may involve important structures such as the sympathetic chain, contents of the carotid sheath, and branches of the hypoglossal, lingual, and facial nerves [18].

There are only very few case reports of late presentation of cystic lymphatic malformations in the neck. A case was reported by El Sayed et al. [19] of a case of cystic hygroma appearing in the posterior triangle of the neck at 12 years and extending into the mediastinum.

MRI is recommended for all suspected cases of lymphatic malformations. It offers accurate preoperative staging, which is essential for identifying individual cystic areas and assessing the extent of involvement of adjacent structures, particularly nerves and blood vessels. This information is highly valuable for surgical planning [20].

If a mass appears following an upper respiratory tract infection, reactive lymphadenopathy is the most likely cause, though a secondary infected congenital cystic neck mass is also a close differential diagnosis.

Cystic lymphatic malformations typically present as painless swellings, while cervical lymphadenitis is usually tender. Infection or intracystic hemorrhage in a cystic hygroma can lead to tenderness. Although lymphatic malformations are usually soft and fluctuant, bleeding within the cyst may make them firm, tense, and painful. Another distinguishing feature is transillumination, which may be dull in the presence of infection or bleeding. The overlying skin in lymphatic malformations is generally normal, whereas in cervical lymphadenitis, it can appear red, warm, and erythematous.

Traditional management of lymphatic malformations involves surgical removal, aiming to excise affected tissue while preserving vital structures. Because of the complex anatomy of the head and neck, surgical removal can carry cosmetic and functional risks. These lesions often recur, in part because complete excision is frequently not possible. Resection may be beneficial for complex lymphatic malformations, but proper staging is necessary. These operations can be lengthy and require careful dissection to protect important structures [19].

Most recent biomedical literature supports the effectiveness of nonsurgical treatment for lymphatic malformations using percutaneous sclerotherapy with agents such as bleomycin or OK-432. The choice of treatment for lymphatic malformations depends on the lesion's extent, symptoms, and location. Focal and macrocystic lymphatic malformations can be treated with either sclerotherapy or surgical removal, while diffuse and predominantly microcystic lymphatic malformations are more challenging to eliminate. Ablation or resection is indicated for recurrent infections, cosmetic concerns, deformity, dysfunction, or fluid leakage. Recurrence after apparently complete surgical excision ranges from 15%-40%, often due to regrowth of residual lymphatic channels. Sclerotherapy in the residual cavity after excision may reduce recurrence [20].

If sudden enlargement and pain in a lymphatic malformation are caused by bleeding within the lesion, conservative management with rest and pain relief is usually adequate. Enlargement associated with systemic viral or bacterial infections can also be managed expectantly, as it is generally benign. However, bacterial infections that present with cellulitis require prompt intervention. Infected lymphatic malformations become tense and swollen and may cause redness, pain, and systemic symptoms; this complication occurs in about 15%-20% of cases. Treatment involves systemic antibiotics, and hospitalization for intravenous therapy is often required [16].

## Conclusions

Cystic lymphatic malformations should be considered a rare but important differential diagnosis for sudden-onset neck swelling in the posterior triangle. Clinical features such as site, appearance, consistency, tenderness, and transillumination may be unreliable in reaching an accurate diagnosis. Radiological investigations are often crucial in distinguishing between cervical lymphadenitis and lymphatic malformations. Clinical examination, along with MRI imaging, can reliably confirm cystic lymphatic malformation. MRI is valuable for preoperative planning and for assessing the extent of the lesion. Management typically involves surgical excision with careful dissection to preserve vital structures, or alternatively, nonsurgical approaches such as percutaneous sclerotherapy with agents like bleomycin or OK-432. The indications for treating lymphatic malformations depend on the extent, symptoms, and anatomical location of the lesions.
